# USING HEALTH INFORMATION FROM AFFORDABLE WEARABLE DEVICES FOR PREDICTING IMPENDING TIPPING POINTS

**DOI:** 10.1093/geroni/igz038.2220

**Published:** 2019-11-08

**Authors:** Jian Liu, Beverly Heasley, Janice D Crist, kimberly D Shea, Lorraine M Martin Plank, Linda R Phillips, Rachel L Peterson

**Affiliations:** 1 University of Arizona, Tucson, Arizona, United States; 2 University of Arizona College of Nursing, tucson, Arizona, United States; 3 University of Arizona College of Public Health, Tucson, Arizona, United States

## Abstract

It is desirable to assess older adults’ health status from their routine activities, identify tipping points based on quantitative metrics, and evaluate the potential risks. Such assessments based on infrequently measured, traditional physical performance instruments fall short of sensitivity as well as reliability. In this research, we investigate multiple types of data collected continuously from older adults wearing fitness watches, which are equipped with various wearable wireless sensors. We develop a methodology to fuse the information from multivariate data streams and define a new synthesized metric to detect significant physiological transitions that lead to tipping points. Both sensitivity- and robustness-analysis are conducted to evaluate the risks of miss-detection and false alarm. The detection results are cross-validated by the self-reported data from daily diaries and questionnaires.

